# Insomnia

**Published:** 1890-03

**Authors:** 


### INSOMNIA.

Insomnia is rightly regarded as one of the marks of an overwrought or worried nervous system, and conversely we may take it that sound sleep, lasting for a reasonable period—say from six to nine hours in the case of adults—is a fair test of nervous competence. Various accidental causes may temporarily interfere with sleep in the healthy; but still the rule holds good, and a normal brain reveals its condition by obedience to this daily rhythmic variation. Custom can do much to contract one's natural term of sleep, a fact of which we are constantly reminded in these days of high pressure; but the process is too artificial to be freely employed. Laborious days, with scanty intervals of rest, go far to secure all the needful conditions of insomnia. In allotting hours of sleep it is impossible to adopt any maxim or uniform custom. The due allowance varies with the individual. Age, constitution, sex, fatigue, exercise, each has its share of influence. Young persons and hard workers naturally need and should have more sleep than those who neither grow nor labor. Women have by common consent been assigned a longer period of rest than men, and this arrangement, in the event of their doing hard work, is in strict accord with their generally lighter physical construction and recurrent infirmities. Absolute rule there is none, and it is of little moment to fix an exact average allowance provided the recurrence of sleep be regular and its amount sufficient for the needs of a given person, so that fatigue does not result in such nerve prostration and irritability as render healthy rest impossible.—*London Lancet.*

